# Possible Role of Mitochondrial Transfer RNA Gene 5816 A > G Genetic Polymorphism (m.5816A > G) in a 3-Year-Old Child with Dystonia: Report of a Case

**DOI:** 10.1055/s-0043-1774708

**Published:** 2023-09-27

**Authors:** Sumei Wang, Minglu Liang, Jiehui Ma, Sheng Huang, Lili Fan, Feng Zhu, Dan Sun

**Affiliations:** 1 Department of Pediatric Neurology, Wuhan Children's Hospital, Tongji Medical College, Huazhong University of Science and Technology, Wuhan, Hubei, China; 2 Clinic Center of Human Gene Research, Union Hospital, Tongji Medical College, Huazhong University of Science and Technology, Wuhan, Hubei, China

**Keywords:** mitochondrial DNA mutation, mitochondrial diseases, dystonia, epilepsy

## Abstract

**Background** Mutations in the mitochondrial transfer RNA (mt-tRNA) gene are a hotspot for mitochondrial DNA (mtDNA) mutations and are most common in mitochondrial diseases.

**Methods** We identified the mt-tRNA gene 5816 A > G (m.5816 A > G) mutation in a 3-year-old child with dystonia who died. We performed clinical evaluation, genetic analysis, and biochemical investigation with mitochondrial function testing.

**Results** Our patient was found to have dystonia with hyperlactatemia. Electroencephalogram findings were abnormal in children with numerous multifocal spikes, multispike, spikes and slow waves, slow waves and low amplitude fast waves, more pronounced in the occipital region bilaterally, and occurring continuously during sleep. One year later, the preexisting patient had seizures lasting 1 to 2 hours and subsequently died. mtDNA sequencing revealed that the proband, her mother, and her grandmother all carried the m.5816A > G mutation. Oxygen consumption rate (OCR) assays revealed that the proband's basal resting OCR, adenosine triphosphate production, proton leak, maximal respiration, and spare capacity OCR were all significantly lower compared with healthy children of the same age.

**Conclusion** The present case demonstrates a childhood dystonia caused by a mt-tRNA gene 5816 A > G mutation, which has never been reported before. Our findings provide valuable new insights into the pathogenic mechanism and function of the m.5816A > G mutation.

## Introduction

Dystonia is a group of movement disorders characterized by distorted, repetitive, abnormal movements, and postures caused by involuntary, sustained muscle contractions. 1 Depending on the etiology, dystonia can be classified as acquired, genetic, or idiopathic. 2 Previous studies have reported several genes associated with primary dystonia, but a proportion of dystonia is secondary to trauma, infection, and inherited neurodegenerative diseases such as Wilson's disease 3 or Huntington's disease. 4

Mitochondria are key organelles for the generation of cellular energy. Mitochondrial diseases are a large group of clinically and genetically heterogeneous disorders caused by mitochondrial dysfunction. 5 6 Mitochondrial dysfunction affects organs with high energy demands, especially muscles, as well as the brain. Primary mitochondrial diseases are defined as pathogenic mutations in mitochondrial DNA or nuclear DNA (mtDNA or nDNA, respectively), usually affecting proteins involved in oxidative phosphorylation (OXPHOS), and include Friedreich's ataxia, Leber's hereditary optic neuropathy, mitochondrial encephalomyopathy, lactic acidosis, and stroke-like episodes (MELAS) syndrome, and myoclonic epilepsy with ragged red fibers. 7 Pathogenic mutations can induce different types of deleterious phenotypes manifested as neurodevelopmental delay, seizures, blindness, hearing loss, stroke, and premature death. 8

In this study, we report a case of an infant with dystonia who died with the 5816 A > G mutation in the mitochondrial transfer RNA (mt-tRNA) gene carried by both her mother and maternal grandmother. This case is the first child in China to carry the 5816 A > G mutation in the mt-tRNA gene. We also found that the mitochondrial OXPHOS function was significantly decreased in the primary cells of the previous patients carrying this mutation.

## Methods

### Ethics Compliance

The study was approved by the Ethics Committee of Wuhan Children's Hospital (ethics number: 2022R093-F02). Informed written consent was obtained from all participants and the parents of the proband.

### Whole Exome Sequencing

Genomic DNA samples were extracted from peripheral blood using QIAamp Blood Mini Kit (Qiagen, Hilden, Germany). Quality of genomic DNA was evaluated by agarose gel analysis and quantity was measured by NanoDrop2000 and Qubit3.0. DNA is sheared with M220 Focused-ultrasonicator (Covaris, Woburn, Massachusetts, United States). DNA target regions were captured by hybridizing the genomic DNA sample library with the xGen Exome Research Panel v1.0 (IDT, United States). The captured and amplified DNA samples were sequenced using Illumina NovaSeq6000 (Illumina, San Diego, California, United States) with 150 base-paired end reads.

### Mitochondrial Genome Sequencing and Analysis

Genome was extracted from urine and blood samples using QIAamp DNA Micro Kit and TLANamp Blood DNA Kit, respectively. TaKaRa LA Taq Hot Start Version was used to amplify the full-length mitochondrial genome using the following amplification procedure: 95°C for 2 minutes, 95°C for 20 seconds, and 68°C for 18 minutes (30). After fragmentation of the mitochondrial genome by Covaris M220, the library was constructed using NanoPrepDNA kit (for Illumina). The size of nucleic acid fragments in the library was checked by Agilent 2100 to ensure that the fragment size was around 260 to 400 bp.

Next, bioinformatics analysis was performed by public software and packages. First, preprocessing of reads was carried out using fastp to remove low-quality reads. 9 Second, the Burrows–Wheeler Aligner tool 10 and default parameters were compared with the revised Cambridge Reference Sequence, 11 the generated BAM file, sorted by SAMtools 10 ; finally, VarDict 12 detected single nucleotide variant (SNV) and indels (<50 bp), and ANNOVAR 13 and MSeqDR 14 annotate the database for SNV.

### Primary Skin Fibroblast Cell Culture

The patient's skin on the medial side of the large arm is first disinfected and locally anesthetized. The loop drill is placed perpendicular to the skin and then pressed firmly downward while rotating. The core area was fully elevated with forceps or the tip of a needle, and a small amount of tissue mass was excised at the bottom of the core area with small scissors and immediately placed in phosphate buffer. The tissue mass was cut into 1 mm three pieces and added to DMEM complete medium containing 15% fetal bovine serum, 1% penicillin and streptomycin, and incubated at 37°C and 5% CO _2_ . When skin fibroblasts grew around the tissue block (usually about 1 week), 0.25% trypsin was added to digest the cells, and then 1 mL of culture medium was added and cultured at 37°C and 5% CO _2_ .

### Cellular Oxygen Consumption Rate Assay

Primary skin fibroblasts were inoculated into XF24 cell culture microtiter plates (5 × 104 cells/well). Prior to testing, the medium was changed to specific XF assay medium (pH 7.4) and the cells were allowed to stand for 1 hour at 37°C in a CO _2_ -free incubator. According to the instructions, oligomycin (2 μM), trifluoromethoxy carbonylcyanide phenylhydrazone (2 μM), rotenone (1 μM), and antimycin (1 μM) were prepared and added to the wells of the assay plate. The test plates were placed on the pallets of the Seahorse XFe24 Extracellular Flux Analyzer (Agilent Technologies Ltd., Santa Clara, California, United States) and the oxygen consumption rate (OCR) was measured three times (3 minutes/time).

## Results

### Clinical Phenotype of the Proband

In April 2021, the presentee (2 years and 10 months old) had episodes of vomiting during sleep at night without apparent cause, and the vomit consisted of gastric contents of medium volume; 1 hour after vomiting, she had decreased consciousness response, did not respond to calls, eyes were closed, mouth twitched, and she had been given bath, and her limbs continued to shake for about 1 hour, without fever or diarrhea; her parents urgently took her to a hospital near her place of residence (the specific treatment strategy was not clear), and her symptoms subsided. In August 2021, while sleeping at night, the subject again vomited several times, followed by salivation, closed eyes, and reluctance to speak, and her symptoms subsided after 1 hour. After the symptoms subsided, the patient was very tired and was again taken to a hospital near her home for treatment (the exact treatment strategy was not known). After being discharged from the hospital, the patient gradually developed abnormal behavior: reluctance to play with other children, reluctance to speak, slurred speech, choking on water, poor sleep, noisy at night, unstable walking, easy to fall, and gradually increasing symptoms.

The preexisting patient was admitted to Wuhan Children's Hospital on September 21, 2021, for further definitive diagnosis and treatment. The mother reported that she had one pregnancy and one delivery, and the perinatal period was safe and uneventful

l without hypoxic asphyxia. The mother had symptoms of double eyelid blinking and was diagnosed with possible epilepsy by the hospital near her home and was treated with oral oxcarbazepine. The grandmother had hand tremors.

Physical examination of the patient revealed a delirious state of confusion and poor mental responsiveness. She had limited abduction of the left eye, along with salivation, irritability and agitation, unsteady gait, and persistent hand tremors. The proband had unrestricted limb movement, normal muscle tone, normal bilateral knee tendon reflexes, and both Babinski, cervical resistance, and Kerning signs were negative. Electrocardiogram, five-category blood count, parathyroid hormone, blood ammonia, liver function, renal function, cardiac enzyme profile, electrolytes, lipids, blood glucose, immune panel, 25-hydroxyvitamin D, erythrocyte sedimentation rate, calcitoninogen, and high-sensitivity C-reactive protein were all normal. Cerebrospinal fluid was negative for autoimmune-related antibodies.

**Fig. 1:**
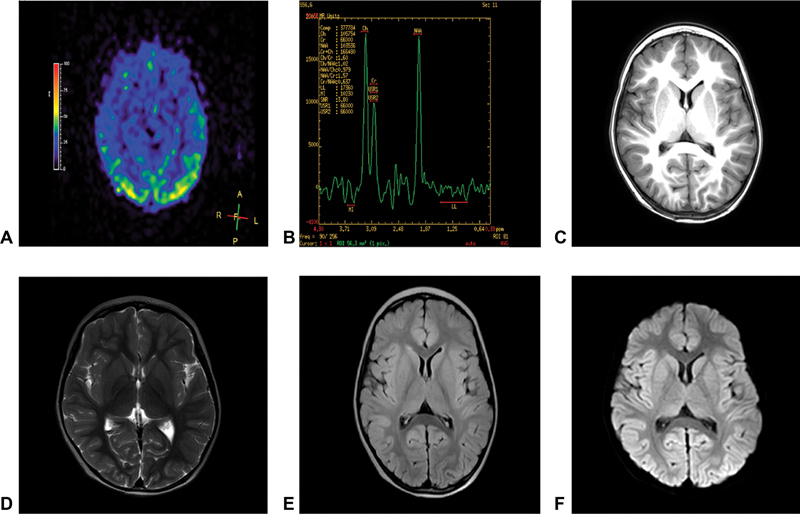
The magnetic resonance imaging of the proband. ( **A** ) ASL, ( **B** ) MRS, ( **C** ) T1, ( **D** ) T2, ( **E** ) T2 fluid-attenuated inversion recovery, and ( **F** ) diffusion-weighted imaging.

The electroencephalogram (EEG) results were abnormal in the children with a large number of multifocal spikes, multispikes, spikes and slow waves, slow waves and low amplitude fast waves, more pronounced in the occipital region bilaterally, and occurring continuously during sleep. The cranial magnetic resonance imaging (MRI) scan showed no abnormal signal in the brain parenchyma, and the arterial spin labeling (ASL) suggested a symmetrical increase in perfusion in the bilateral parieto-occipital lobes; the cranial MRI + diffusion-weighted imaging scan showed no obvious abnormal signs ( Fig. 1 ).

The proband was admitted to our hospital for treatment several times between September 2021 and May 2022. During this period, the proband's multiple EEG findings suggested persistent bilateral occipital discharges. We adjusted the treatment for dystonia, including benzhexol, levodopa, and clonazepam, but the results were poor. Throughout the course of the disease, the patient's lactate level was higher than normal, and the results are shown in Table 1 . At the end of May 2022, during the final course of the disease, the previous patient developed a convulsive state with convulsions lasting 1 to 2 hours, manifested by double-eye gaze, cyanosis of the lips, rhythmic shaking of the extremities, and profuse salivation, and then died.

**Table 1:** The lactate level of proband

Time	September 27, 2021	February 7, 2021	December 12, 2021	January 11, 2022	February 3, 2022	March 22, 2022	June 1, 2022	Reference (mmol/L)
Lactate	**4.04**	**2.68**	**2.54**	2.10	**2.32**	1.77	**2.63**	0.5–2.22

Note: bold signifies reference values higher than (2.22).

### Sequencing

**Fig. 2:**
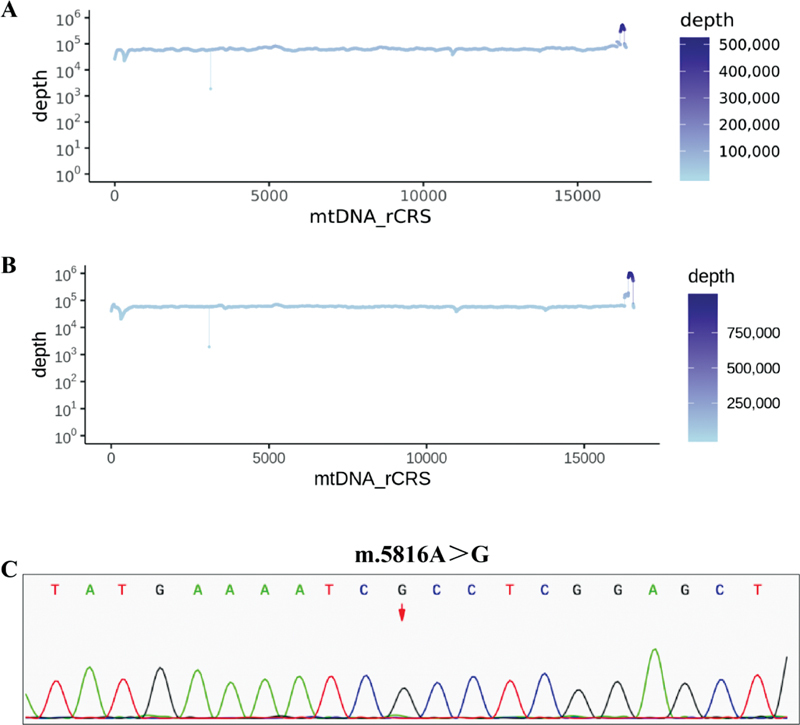
Sequence chromatogram confirming the m.5816A > G mutation in urine and blood from proband. ( **A** ) rCRS of blood, ( **B** ) rCRS of urine, and ( **C** ) sequence of m.5816A > G. mtDNA, mitochondrial DNA; rCRS, revised Cambridge Reference Sequence.

**Fig. 3:**
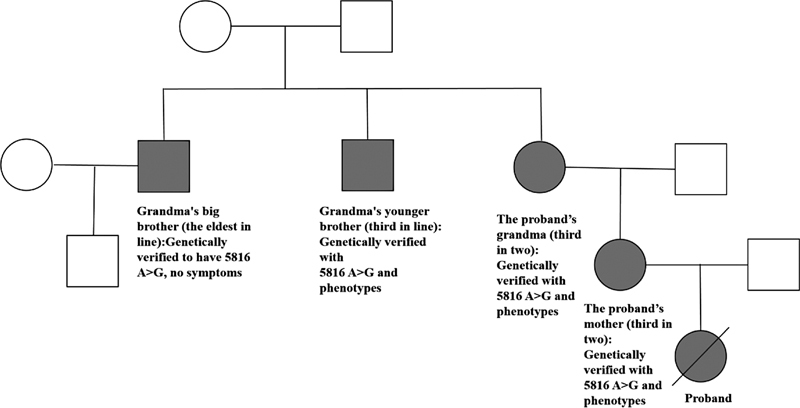
The family tree of proband showing all affected family members known to harbor the m.5816A > G mutation (closed symbols).

In October 2021, we first performed whole-exome and copy number variant sequencing of the blood of the proband; however, no pathogenic or potentially pathogenic variants highly correlated with the clinical presentation of the patient were identified. Considering that both the mother and grandmother of the proband had symptoms of epilepsy, we obtained blood and urine samples from the proband for mitochondrial genome sequencing. The results are shown in Fig. 2 , where the proband carried a novel mutation m.5816A > G. In addition, we also sequenced the mitochondrial genomes of the blood and urine samples from the mother and grandmother of the proband. The results showed that both the mother and grandmother carried m.5816A > G. The family tree of the proband is shown in Fig. 3 .

### OCR to Detect Changes in Mitochondrial Function

**Fig. 4:**
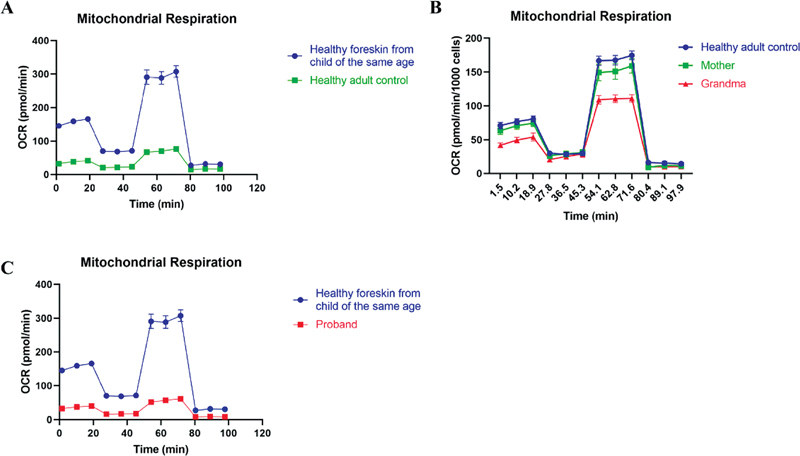
The oxygen consumption rate (OCR) experiments of ( **A** ) healthy child, proband, ( **B** ) her mother, and ( **C** ) her grandmother.

Next, we obtained primary cells from the proband, her mother, and grandmother, and performed OCR experiments to observe the changes in mitochondrial function in the cells after the m.5816A > G mutation. Fig. 4A shows that the function of the mitochondrial respiratory chain was more vigorous in the primary cells of children compared with adults. Fig. 4B shows that there was no significant change in mitochondrial respiratory chain function in progenitor cells from the proband's mothers and grandmothers compared with adults who did not carry the m.5816A > G mutation; however, the progenitor cells from the proband had only 28.6% basal OCR at rest compared with healthy children of the same age who did not carry the m.5816A > G mutation ( Fig. 4C ). We also found that adenosine triphosphate (ATP) production, proton leak, maximal respiration, and reserve capacity OCR were reduced to 28.6, 20.1, 24.1, and 11.1%, respectively, in the progenitor cells.

## Discussion

This case is the first Chinese family with a mutation in the mitochondrial m.5816A > G locus. In this case study, the proband was diagnosed with dystonia. The EEG findings showed a large number of multifocal spikes, multispike, spikes and slow waves, and slow waves and low amplitude fast waves bilaterally in the posterior head. The proband's mother and grandmother also had partial epilepsy symptoms. Mitochondrial genomic analysis revealed the presence of the m.5816A > G mutation in the mitochondrial tRNA (MTT) gene, and both her mother and grandmother carried this mutation. We also analyzed the mitochondrial OXPHOS function in the primary cells of the proband, her mother, and her grandmother. Interestingly, the mitochondrial OXPHOS function was significantly decreased in the primary cells of the proband, whereas no significant changes were observed in her mother and grandmother.

Mitochondria are important organelles in cells and their main function is to produce ATP to supply energy to the cell. The distribution of mitochondria in cells from different tissues depends on energy requirements. Mitochondrial dysfunction can affect several tissues, particularly those with high energy demands, especially the brain. Compared with other tissues, the brain consumes almost 10 times more oxygen and glucose. Given the high demand and consumption of ATP in the brain, most mitochondrial mutations affect brain function and lead to neurological pathologies. 15

Mitochondrial diseases are clinically heterogeneous disorders characterized by mitochondrial dysfunction, 16 17 mainly caused by Mendelian inherited mutations in nDNA and/or maternally inherited mtDNA. 18 19 Mutations in mtDNA are found in most mitochondrial diseases, and most of these mutations are located in the mt-tRNA gene (MTT gene). 20 MTT gene is essential for protein synthesis, is a hotspot for mtDNA mutations, and leads to involvement of tissues and organs with greater aerobic metabolism. 21 Today, more than 250 MTT mutations causing disease have been reported. 22 The same tRNA mutation may lead to different mutational loads and various types of clinical manifestations, ranging from asymptomatic to severe phenotypes. 23 For example, the m.1A > G mutation in MTT gene 3243 causes MELAS syndrome, chronic progressive external oculomotor paralysis, maternally inherited diabetes mellitus and deafness syndrome, and two or more of these phenotypes may occur together in the same family. 24 In addition, Scuderi et al reported another adjacent mutation (m.5814T > C) that disrupts Watson–Crick base pairing in the D-stem and is associated with mitochondrial encephalopathy. 25

**Fig. 5:**
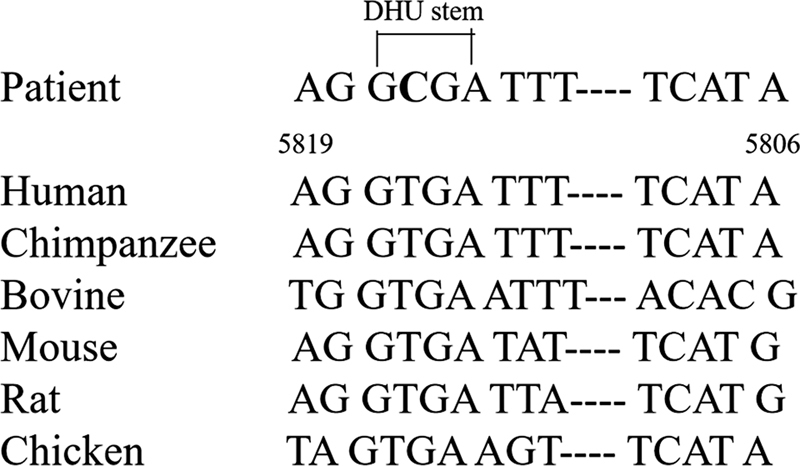
Evolutionary conservation of the T-A base pairing in the dihydrouridine (DHU) stem of mt-tRNACys.

**Fig. 6:**
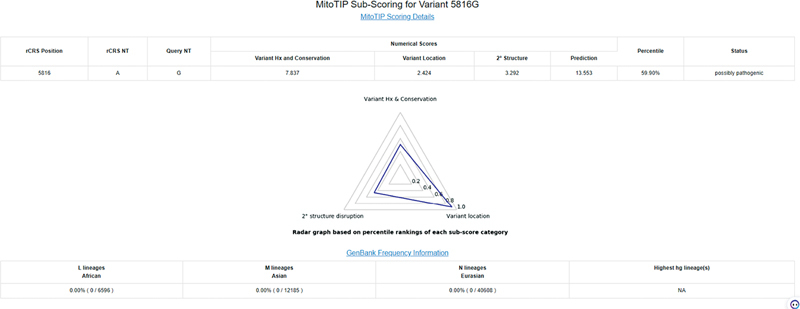
The prediction of pathologic finding for m.5816A > G.

Fewer studies have been conducted on mutations at the m.5816A > G locus, which shows a high degree of evolutionary conservation ( Fig. 5 ). We used the mtDNA polymorphic variant database MITOMAP ( https://www.mitomap.org/cgi-bin/mitotip?pos=5816&alt=G&quart=2 ) to predict the pathogenicity of the m.5816A > G locus mutation, and the results showed that the m.5816A > G locus mutation was pathogenic with a probability of 59.9% ( Fig. 6 ). 26 The possible reasons for this are, first, that the structural integrity of mt-tRNACys is essential for maintaining the helical conformation as well as the interactions between the translation components. 27 Therefore, the m.5816A > G mutation may disrupt the mt-tRNACys structure and affect Watson–Crick base pairing in the dihydrouridine arm of mt-tRNACys. 28 Second, the abnormal structure may lead to a significant decrease in the steady-state level of mt-tRNACys and consequently to a mitochondrial translation defect and subsequent OXPHOS dysfunction. 23 Both of these points support the deleterious nature of the m.5816A > G locus mutation.

Data from the OCR assay showed a significant decrease in ATP production and maximal respiration in the cells of the proband compared with healthy children of the same age who did not carry the m.5816A > G mutation, accompanied by an increase in serum lactate, suggesting that OXPHOS in the mitochondria may be severely impaired. However, it is interesting to note that ATP production and maximal respiration were not significantly reduced in the cells of the proband's mother and grandmother. More surprisingly, mitochondrial ATP production, proton leak, maximal respiration, and OCR reserve capacity were much higher in healthy children than in normal adults ( Fig. 4A ). We hypothesized that the mitochondrial OXPHOS function is more vigorous in children compared with adults due to their growth and developmental stage. In this case, the proband had an onset at 3 years and 3 months of age and died less than a year later, while her mother and grandmother had no life-threatening symptoms and only partial epilepsy. McFarland et al reported a family in which progressive dystonia in family members was also caused by the m.5816A > G mutation. The patients in this case were all adults and adolescents with good survival. Therefore, we speculate that the m.5816A > G mutation is likely to cause severe mitochondrial dysfunction, which is more harmful in children.

There are several limitations to this report. First, we only obtained blood and urine from the subject, mother, and grandmother for mitochondrial genome sequencing, and further serial muscle sections should be performed to clarify the pathological features of mitochondrial disease; second, multiple perspectives are needed to evaluate the mitochondrial dysfunction caused by the m.5816A > G mutation, especially tRNA-Cys steady-state level, mitochondrial protein expression, mitochondrial ROS level, etc. This study will encourage us to combine mitochondrial genome sequencing approaches with clinical symptoms to facilitate accurate genetic diagnosis and correct pathogenicity attribution of childhood dystonia, and to provide appropriate genetic counseling and transmission prevention techniques.

## References

[1] Albanese A Bhatia K Bressman S B Phenomenology and classification of dystonia: a consensus update Mov Disord 2013 28 07 863 873 23649720 10.1002/mds.25475 PMC3729880

[2] di Biase L Di Santo A Caminiti M L Pecoraro P M Carbone S P Di Lazzaro V Dystonia diagnosis: clinical neurophysiology and genetics J Clin Med 2022 11 14 4184 35887948 10.3390/jcm11144184 PMC9320296

[3] Svetel M Kozić D Stefanova E Semnic R Dragasevic N Kostic V S Dystonia in Wilson's disease Mov Disord 2001 16 04 719 723 11481698 10.1002/mds.1118

[4] Ross C A Polyglutamine pathogenesis: emergence of unifying mechanisms for Huntington's disease and related disorders Neuron 2002 35 05 819 822 12372277 10.1016/s0896-6273(02)00872-3

[5] Mattman A Sirrs S Mezei M M Salvarinova-Zivkovic R Alfadhel M Lillquist Y Mitochondrial disease clinical manifestations: an overview B C Med J 2011 53 04 183 187

[6] Ng Y S Turnbull D M Mitochondrial disease: genetics and management J Neurol 2016 263 01 179 191 26315846 10.1007/s00415-015-7884-3 PMC4723631

[7] MitoCanada Mitochondrial disease [Internet] MitoCanada 2022[cited January 27, 2022]. Available at:https://mitocanada.org/mitochondrial-disease/

[8] North American Mitochondrial Disease Consortium Rosales X Q Thompson J LP Haas R The North American mitochondrial disease registry J Transl Genet Genom 2020 4 02 81 90 32601614 10.20517/jtgg.2020.12 PMC7323997

[9] Chen S Zhou Y Chen Y Gu J fastp: an ultra-fast all-in-one FASTQ preprocessor Bioinformatics 2018 34 17 i884 i890 30423086 10.1093/bioinformatics/bty560 PMC6129281

[10] 1000 Genome Project Data Processing Subgroup Li H Handsaker B Wysoker A The sequence alignment/map format and SAMtools Bioinformatics 2009 25 16 2078 2079 19505943 10.1093/bioinformatics/btp352 PMC2723002

[11] Bandelt H J Kloss-Brandstätter A Richards M B Yao Y G Logan I The case for the continuing use of the revised Cambridge Reference Sequence (rCRS) and the standardization of notation in human mitochondrial DNA studies J Hum Genet 2014 59 02 66 77 24304692 10.1038/jhg.2013.120

[12] Lai Z Markovets A Ahdesmaki M VarDict: a novel and versatile variant caller for next-generation sequencing in cancer research Nucleic Acids Res 2016 44 11 e108 27060149 10.1093/nar/gkw227 PMC4914105

[13] Wang K Li M Hakonarson H ANNOVAR: functional annotation of genetic variants from high-throughput sequencing data Nucleic Acids Res 2010 38 16 e164 20601685 10.1093/nar/gkq603 PMC2938201

[14] MSeqDR Consortium Participants MSeqDR Consortium participants: Sherri Bale, Jirair Bedoyan, Doron Behar, Penelope Bonnen, Lisa Brooks, Claudia Calabrese, Sarah Calvo, Patrick Chinnery, John Christodoulou, Deanna Church Rosanna Clima, Bruce H. Cohen, Richard G. Cotton, IFM de Coo, Olga Derbenevoa, Johan T. den Dunnen, David Dimmock, Gregory Enns, Giuseppe Gasparre Amy Goldstein, Iris Gonzalez, Katrina Gwinn, Sihoun Hahn, Richard H. Haas, Hakon Hakonarson, Michio Hirano, Douglas Kerr, Dong Li, Maria Lvova, Finley Macrae, Donna Maglott, Elizabeth McCormick, Grant Mitchell, Vamsi K. Mootha, Yasushi Okazaki Aurora Pujol, Melissa Parisi, Juan Carlos Perin, Eric A. Pierce, Vincent Procaccio, Shamima Rahman, Honey Reddi, Heidi Rehm, Erin Riggs, Richard Rodenburg, Yaffa Rubinstein, Russell Saneto, Mariangela Santorsola, Curt Scharfe Claire Sheldon, Eric A. Shoubridge, Domenico Simone, Bert Smeets, Jan A. Smeitink, Christine Stanley, Anu Suomalainen, Mark Tarnopolsky, Isabelle Thiffault, David R. Thorburn, Johan Van Hove, Lynne Wolfe, and Lee-Jun Wong Falk M J Shen L Gonzalez M Mitochondrial Disease Sequence Data Resource (MSeqDR): a global grass-roots consortium to facilitate deposition, curation, annotation, and integrated analysis of genomic data for the mitochondrial disease clinical and research communities Mol Genet Metab 2015 114 03 388 396 25542617 10.1016/j.ymgme.2014.11.016 PMC4512182

[15] Gandhi S Abramov A Y Mechanism of oxidative stress in neurodegeneration Oxid Med Cell Longev 2012 2012 428010 22685618 10.1155/2012/428010 PMC3362933

[16] Annesley S J Fisher P R Mitochondria in health and disease Cells 2019 8 07 680 31284394 10.3390/cells8070680 PMC6678092

[17] Russell O M Gorman G S Lightowlers R N Turnbull D M Mitochondrial diseases: hope for the future Cell 2020 181 01 168 188 32220313 10.1016/j.cell.2020.02.051

[18] Gorman G S Chinnery P F DiMauro S Mitochondrial diseases Nat Rev Dis Primers 2016 2 16080 27775730 10.1038/nrdp.2016.80

[19] Li H Liu D Lu J Bai Y Physiology and pathophysiology of mitochondrial DNA Adv Exp Med Biol 2012 942 39 51 22399417 10.1007/978-94-007-2869-1_2 PMC4706180

[20] Bohnsack M T Sloan K E The mitochondrial epitranscriptome: the roles of RNA modifications in mitochondrial translation and human disease Cell Mol Life Sci 2018 75 02 241 260 28752201 10.1007/s00018-017-2598-6 PMC5756263

[21] Ng Y S Bindoff L A Gorman G S Mitochondrial disease in adults: recent advances and future promise Lancet Neurol 2021 20 07 573 584 34146515 10.1016/S1474-4422(21)00098-3

[22] Ruiz-Pesini E Lott M T Procaccio V An enhanced MITOMAP with a global mtDNA mutational phylogeny Nucleic Acids Res 2007 35(Database issue):D823 D828 17178747 10.1093/nar/gkl927 PMC1781213

[23] Lin Y Xu X Wang W A mitochondrial myopathy-associated tRNA ^Ser(UCN)^ 7453G>A mutation alters tRNA metabolism and mitochondrial function Mitochondrion 2021 57 1 8 33279600 10.1016/j.mito.2020.11.015

[24] Yarham J W Elson J L Blakely E L McFarland R Taylor R W Mitochondrial tRNA mutations and disease Wiley Interdiscip Rev RNA 2010 1 02 304 324 21935892 10.1002/wrna.27

[25] Scuderi C Borgione E Musumeci S Severe encephalomyopathy in a patient with homoplasmic A5814G point mutation in mitochondrial tRNACys gene Neuromuscul Disord 2007 17 03 258 261 17241783 10.1016/j.nmd.2006.11.006

[26] Brandon M C Lott M T Nguyen K C MITOMAP: a human mitochondrial genome database–2004 update Nucleic Acids Res 2005 33(Database issue):D611 D613 15608272 10.1093/nar/gki079 PMC540033

[27] Carter C W Jr Wolfenden R tRNA acceptor-stem and anticodon bases embed separate features of amino acid chemistry RNA Biol 2016 13 02 145 151 26595350 10.1080/15476286.2015.1112488 PMC4829288

[28] McFarland R Chinnery P F Blakely E L Homoplasmy, heteroplasmy, and mitochondrial dystonia Neurology 2007 69 09 911 916 17724295 10.1212/01.wnl.0000267843.10977.4a

